# Mapping Research Trends and Hotspots in the Link between Alzheimer’s Disease and Gut Microbes over the Past Decade: A Bibliometric Analysis

**DOI:** 10.3390/nu15143203

**Published:** 2023-07-19

**Authors:** Ruipu Xiu, Qingyuan Sun, Boya Li, Yanqing Wang

**Affiliations:** Department of Physiology, School of Basic Medical Sciences, Cheeloo College of Medicine, Shandong University, Jinan 250012, China

**Keywords:** gut microbiota, Alzheimer’s disease, bibliometric analysis, hotspots, neuroinflammation

## Abstract

Alzheimer’s disease (AD) is a globally prevalent neurodegenerative disorder, the underlying causes and mechanisms of which remain elusive. The emerging interest in the potential connection between gut microbes and AD prompted our study to investigate this field through bibliometric analysis. To examine research trends over the past decade, we collected relevant data using search terms associated with gut microbiota and AD from the Web of Science Core Collection. Our analysis involved various tools, including R (version 4.2.2), VOSviewer (version 1.6.18), CiteSpace software (version 6.2.R1), and an online bibliometric platform. Our findings identified a total of 1170 articles published between 2012 and 2022, indicating a consistent growth of research interest in this area. Notably, China significantly contributed with 40.7% (374) of the publications, signifying its prominent role in this field. Among the journals, the Journal of Alzheimer’s Disease published the highest number of articles (57; 4.9%). In terms of author influence, Wang Y, with an H-index of 13, emerged as the most influential author. Additionally, Shanghai Jiaotong University was the most productive institution, accounting for 66 articles (5.6%). Through keyword analysis, we grouped high-frequency keywords into six clusters: gut microbiota, AD, neuroinflammation, gut-brain axis, oxidative stress, and chain fatty acids. Chain fatty acids, oxidative stress, and the gut-brain axis emerged as dominant research topics in the past five years. Recent studies have specifically focused on “nlrp3 inflammasome” and “clearance” (2020–2022), indicating shifting research interests within this field. This bibliometric analysis aims to provide insights into the evolving landscape of research on the gut microbiota and AD. Our results identify key research trends and themes while highlighting potential research gaps. The findings offer valuable perspectives for future investigations, advancing our understanding of AD and exploring potential therapeutic strategies.

## 1. Introduction

The human gut microbiota is a complex and diverse community that plays a significant role in various systems [1], including the central nervous system (CNS) [2], endocrine system [3], and immune system [4,5]. Recent research has begun to unravel its potential influence on AD, a neurodegenerative disorder that represents the seventh leading cause of death in the United States [6,7]. Despite numerous studies, the etiology of AD remains elusive. However, the gut microbiome has started to emerge as a plausible contributor to the pathophysiology of AD. The microbiota-gut-brain axis, a communication network that connects the CNS with the gut microbiota, is thought to play a significant role in the progression and manifestation of AD [8,9]. Furthermore, the dysbiosis, or imbalance, of the gut microbiota is suspected to affect neuroinflammation and neurodegeneration, two key components of AD’s pathogenesis [10,11]. While the connection between the gut microbiota and the pathophysiology of AD holds promise for novel treatment avenues [12], it becomes paramount to conduct more in-depth research to fully unravel the complexities of this relationship.

With the growing interest in the linkage between gut microbiota and AD, it becomes imperative to examine the research trends and to understand the primary focal points of the studies conducted in this field. In this context, bibliometric analysis becomes an invaluable tool. Bibliometric analysis helps elucidate the contributions and interplay among various research components, thereby highlighting the academic progress in this field. Therefore, we utilized bibliometric analysis, encompassing performance assessments and science mapping, to delve into the research patterns concerning gut microbiota and AD.

Despite the vast body of research on gut microbiota and AD, a comprehensive understanding of the overall research trends and key focus areas in this field remains lacking. This study aims to bridge this gap, providing valuable insights into the intellectual structure, dynamic trends, and prospective hotspots in this domain. Understanding these trends could be instrumental in guiding future research directions and contributing to the development of novel therapeutic interventions for AD. Future studies focusing on the gut microbiota’s modulation and its potential benefits for AD might open the door for microbiota’s modulation and microbiota-based therapies, thus revolutionizing the way we approach, manage, and treat AD [13,14].

## 2. Methods

### 2.1. Data Source and Search Strategy

**Figure 1:**
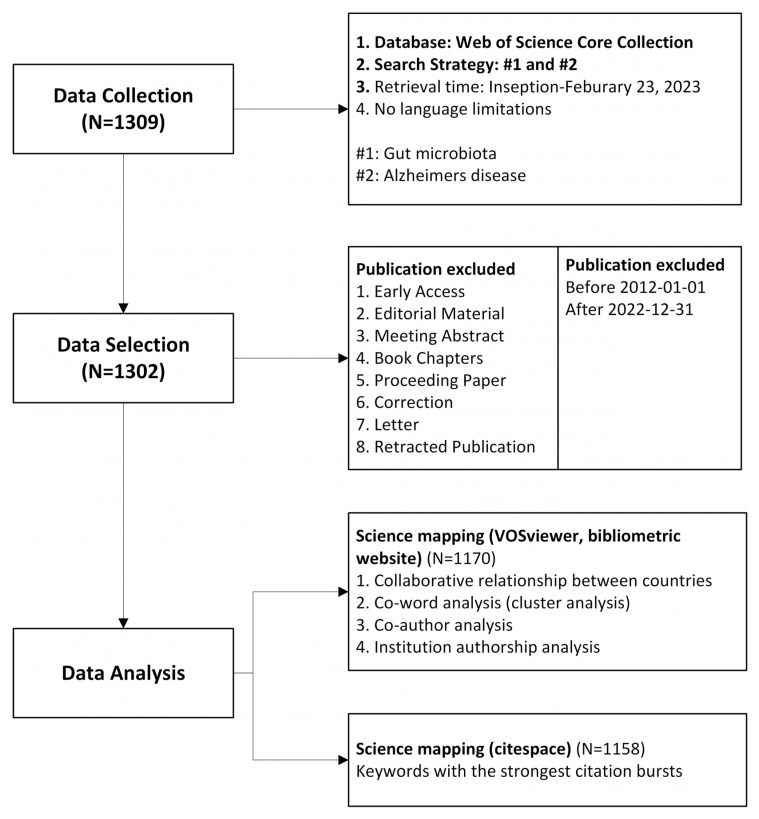
Schematic representation of data collection and study design process.

To collect relevant data, we searched the Web of Science Core Collection (WoSCC) from its inception until 23 February 2023. We used search terms related to gut microbiota and AD, as recommended in previous studies. The search strategy we used was as follows: (“Gastrointestinal Microbiome*” OR “Microbiome, Gastrointestinal” OR “Gut Microbiome” OR “Gut Microbiome*” OR “Microbiome, Gut” OR “Gut Microflora” OR “Microflora, Gut” OR “Gut Microbiota*” OR “Microbiota, Gut” OR “Gastrointestinal Flora” OR “Flora, Gastrointestinal” OR “Gut Flora” OR “Flora, Gut” OR “Gastrointestinal Microbiota*” OR “Microbiota, Gastrointestinal” OR “Gastrointestinal Microbial Community” OR “Gastrointestinal Microbial Communities” OR “Microbial Community, Gastrointestinal” OR “Gastrointestinal Microflora” OR “Microflora, Gastrointestinal” OR “Gastric Microbiome*” OR “Microbiome, Gastric” OR “Intestinal Microbiome*” OR “Microbiome, Intestinal” OR “Intestinal Microbiota*” OR “Microbiota, Intestinal” OR “Intestinal Microflora” OR “Microflora, Intestinal” OR “Intestinal Flora” OR “Flora, Intestinal” OR “Enteric Bacteria” OR “Bacteria, Enteric”) AND (“Alzheimer Dementia*” OR “Dementia, Alzheimer” OR “Alzheimer’s Disease” OR “Dementia, Senile” OR “Senile Dementia” OR “Dementia, Alzheimer Type” OR “Alzheimer Type Dementia” OR “Alzheimer-Type Dementia (ATD)” OR “Alzheimer Type Dementia (ATD)” OR “Dementia, Alzheimer-Type (ATD)” OR “Alzheimer Type Senile Dementia” OR “Primary Senile Degenerative Dementia” OR “Dementia, Primary Senile Degenerative” OR “Alzheimer Sclerosis” OR “Sclerosis, Alzheimer” OR “Alzheimer Syndrome” OR “Alzheimer* Disease*” OR “Senile Dementia, Alzheimer Type” OR “Acute Confusional Senile Dementia” OR “Senile Dementia, Acute Confusional” OR “Dementia, Presenile” OR “Presenile Dementia” OR “Alzheimer Disease, Late Onset” OR “Late Onset Alzheimer Disease” OR “Alzheimer’s Disease, Focal Onset” OR “Focal Onset Alzheimer’s Disease” OR “Familial Alzheimer Disease* (FAD)” OR “Alzheimer Disease, Familial (FAD)” OR “Alzheimer Disease, Early Onset” OR “Early Onset Alzheimer Disease” OR “Presenile Alzheimer Dementia”). The data were extracted from the included publications and exported in either “Plain text file” or “Tab-delimited file” formats. The data were recorded as “Full record and cited references” to ensure comprehensive coverage. Figure 1 is the visual representation of the data collection process.

### 2.2. Data Analysis

The trends in the research were both qualitatively and quantitatively examined using the R program (version 4.2.2) and CiteSpace software (6.2.R1) [15]. The Bibliometrix package in the R software facilitated the exploration of the traits and patterns within the publications, focusing on aspects such as citation count, source, and authorship [16]. The caliber of publications and journals was evaluated via the h-index and 2021 Impact Factor (IF) [17]. The bibliometric website (https://bibliometric.com/, accessed on 24 February 2023) allowed for a graphical depiction of collaborations between countries. With VOSviewer software (version 1.6.18), science mapping visualizations were created, portraying the cooperative relationships between authors, institutions, and research subjects in the domain of gut microbiota and AD [18]. In these maps, each node, representing an author, an institution, or a keyword, indicated the total number of publications or keyword occurrences, while the connecting edge thickness indicated the association strength between nodes, represented by the Total Link Strength (TLS). In the overlay maps, articles were grouped into different clusters distinguished by colors ranging from blue to yellow, based on the Average Publication Year (APY). CiteSpace software provided a keywords burst detection feature, which helped track the evolution of research hotspots over time and forecast future developments in the field. 

## 3. Results

### 3.1. Distribution of Annual Publications and Citations

**Figure 2:**
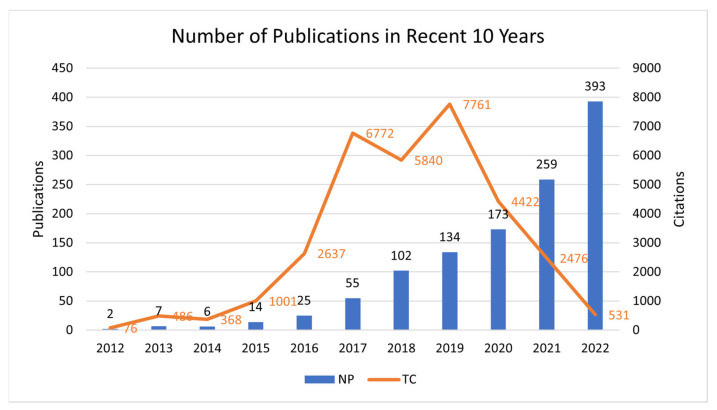
Trends in publications on the relationship between gut microbiota and Alzheimer’s disease, as indicated by the number of publications (NP) and total citation (TC) over time.

Our investigation revealed 1170 publications between 2012 and 2022. As shown in Figure 2, there has been a notable upsurge in the volume of papers on gut microbiota and AD since 2014. This could be due to advancements in research techniques enabling more detailed exploration of gut microbiota, emerging evidence linking gut health to neurological conditions, or a combination of these and other factors. It might also indicate that this area has become a hotbed of research, with many scientists now devoting their efforts towards understanding the link between gut microbiota and AD.

Table 1 provides a more detailed view of these publications, along with their citation counts, which serve as an indicator of their impact and significance in the scientific community. A high citation count typically suggests that a publication has made substantial contributions to the field, as it has been frequently referenced by other researchers in their work.

### 3.2. Geographical Distribution

**Figure 3:**
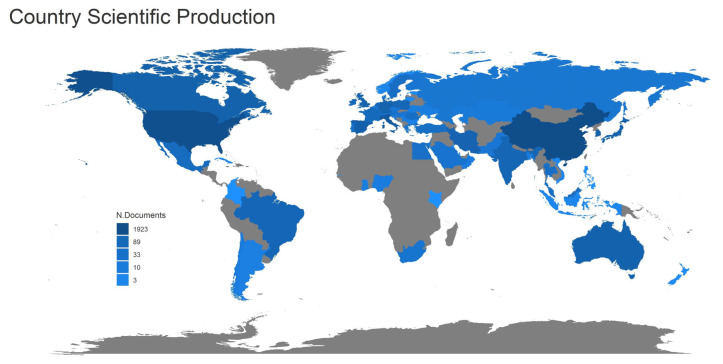
Global distribution of publications on the relationship between gut microbiota and Alzheimer’s disease. The absence of any publication from these countries is represented by the color gray.

A total of 67 countries worldwide have made contributions to publications on gut microbiota and AD. The top ten contributors are China (1846; 27.9%), the USA (1427; 21.5%), Italy (473; 7.1%), Spain (258; 3.9%), Japan (190; 2.9%), Canada (189; 2.9%), Australia (176; 2.7%), Germany (173; 2.6%), and the UK (163; 2.5%), with China and the USA leading the pack (Figure 3). The results indicate a wide interest and global efforts in the field. China and the USA stand out as the most significant contributors, producing approximately 50% of the total publications combined. This suggests that these two countries are likely heavily investing in this research field, probably due to the high prevalence of AD and the potential impact that research findings in this area could have on their population’s health.

**Figure 4:**
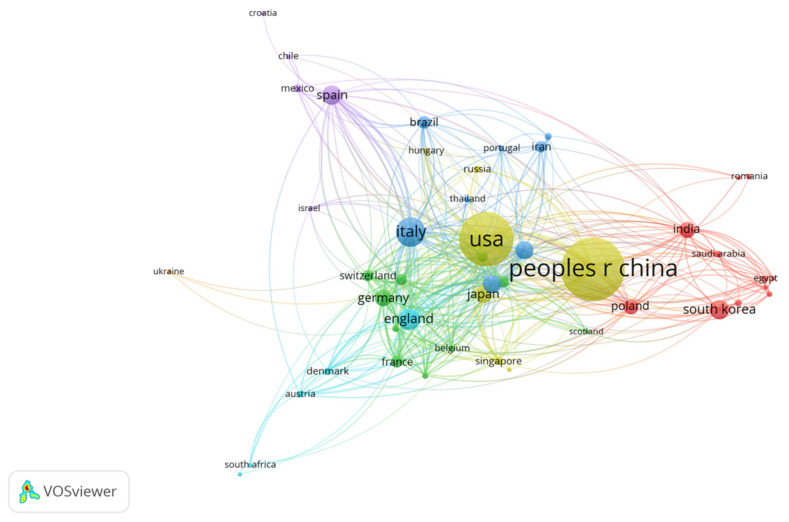
Map of international collaboration networks in research on gut microbiota and AD. Clusters are represented by dots of distinct colors, symbolizing varying levels of cooperation among countries with each cluster. China and the United States have the highest number of publications and the closest collaboration, while a closer network of collaboration is observed between the USA and European countries.

The collaborative network among these countries, shown in Figure 4, shows that the most frequent collaborations are between China and the USA, followed by the USA with Canada and Ireland. This underlines the importance of international cooperation in enhancing scientific understanding in this complex field.

When only corresponding authors are taken into account, 63 countries have contributed to publications on gut microbiota and AD. The top three contributors, as shown in Table 2, are China (374; 40.7%), the USA (208; 22.6%), and Italy (90; 9.8%), with the USA having the most citations (9544). It is interesting to note that the top three contributors remain the same (China, USA, Italy), but the USA leads in terms of citation count. This might suggest that the work led by the USA is particularly influential or groundbreaking in this field.

The Multiple Country Publication (MCP) ratio is an indicator of international collaboration. Among the top 10 countries, Canada has the highest MCP ratio (MCP ratio = 0.419), suggesting a strong tendency towards international collaboration in their research efforts. In contrast, South Korea, with the lowest MCP ratio (MCP ratio = 0.080), appears to favor domestic collaborations, indicating a more internally focused research strategy (Table 2). The MCP is shown in Figure S1.

### 3.3. Journals

The 1170 identified publications were published in 433 journals, demonstrating a broad spread of research across multiple platforms and potentially diverse audiences. Table 3 displays the top 10 journals with the most publications in this field. The Journal of Alzheimer’s Disease ranked highest, publishing 57 articles (4.9%), followed by Nutrients (50; 4.3%) and the International Journal of Molecular Sciences (49; 4.2%). The results suggest that they may be key sources for researchers and practitioners interested in the intersection of gut microbiota and AD.

The impact factor (IF) of a journal, which typically reflects the average number of citations to recent articles published in that journal, provides an indication of its influence or prestige in the scientific community. The 2022 IFs of the top 10 journals range from 4.160 to 19.227, suggesting a wide variation in their influence. All of them have a substantial impact, considering the highly specialized nature of the subject matter.

The Journal Citation Reports (JCR) categorize journals into quartiles (Q1–Q4) based on their impact factor, with Q1 being the highest. The fact that half of these top 10 journals are classified as Q1 suggests that they are highly respected within the scientific community and are likely the source of influential and cutting-edge research in the field of gut microbiota and AD. The remaining half being classified as Q2 still represents a strong standing within the community.

### 3.4. Authors and Institutions

A large number of authors and institutions are engaged in this field, with 4683 authors from 1940 institutions identified. Table 4 displays the top 10 most active authors and institutions in this area. Wang Y, with 31 publications (2.7%) and an H-index of 13, had the highest total citations with 770, followed by Zhang X (28; 2.4%; H-index = 13) and Zhang Y (26; 2.2%; H-index = 7).

At the institutional level, Shanghai Jiaotong University (66; 5.6%) was the most productive, followed by the University of Kentucky (60; 5.1%) and Zhejiang University (59; 5.0%). This could indicate a strong research focus or expertise in this field at these institutions.

**Figure 5:**
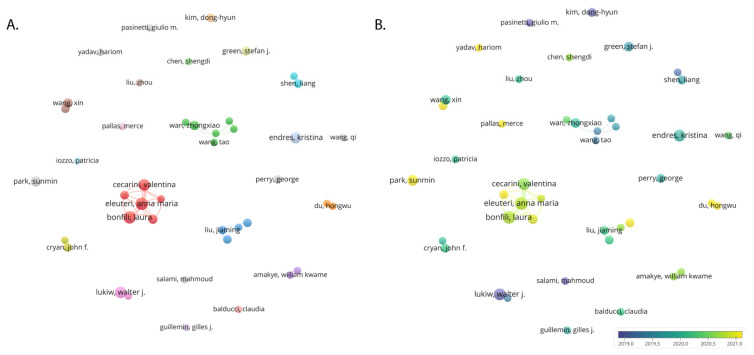
Collaborative networks of co-author analysis, with (**A**) a network visualization map of authors and (**B**) an overlay visualization map of authors. The distribution of authors is more dispersed, indicating a lower degree of collaboration among author groups.

The Total Link Strength (TLS) metric is used to evaluate the intensity of collaborations between researchers. According to the co-author network (Figure 5A), Bonfili L (TLS: 37), Eleuteri AM (TLS: 37), and Cecarini V (TLS: 35) have had the strongest collaborations, which implies that they often work with others and play a central role in the scientific network on gut microbiota and AD. The data from Figure 5B points to Du H (APY: 2022.00; NP: 5; TLS: 2), Liu X (APY: 2021.33; NP: 6; TLS: 3), Gong C (APY: 2021.67; NP: 6; TLS: 25), and Park S (APY: 2021.25; NP: 8; TLS: 0) as the most prolific recent authors in this field, signifying their active engagement and potentially emerging leadership in this area of research. The authors highlighted in yellow represent emerging leaders, significantly contributing to recent developments and advancements in the study of gut microbiota and AD.

### 3.5. Most Cited Articles

Table 5 presents the top 10 most highly cited articles in the field of gut microbiota and AD, suggesting they are particularly influential and have made a significant contribution to the scientific understanding of this area. Citation counts for these articles range from 394 to 1212, demonstrating that they have been frequently referred to in subsequent research, influencing the direction and understanding of the field. This high number of citations is indicative of the recognition and value of these works in the scientific community.

The presence of four articles from Q1 journals with an impact factor (IF) greater than 10 in this top list underlines the importance of these high-impact publications in the field. As stated earlier, journals in the Q1 category are among the most respected and have a high impact factor, suggesting they often contain groundbreaking or highly influential research.

The article with the highest number of citations (N = 1212), “The Microbiota-Gut-Brain Axis”, was published in Physiological Reviews, a top-tier journal with an extremely high impact factor of 46.513.

### 3.6. Keywords

#### 3.6.1. Keywords Co-Occurrence Networks

**Figure 6:**
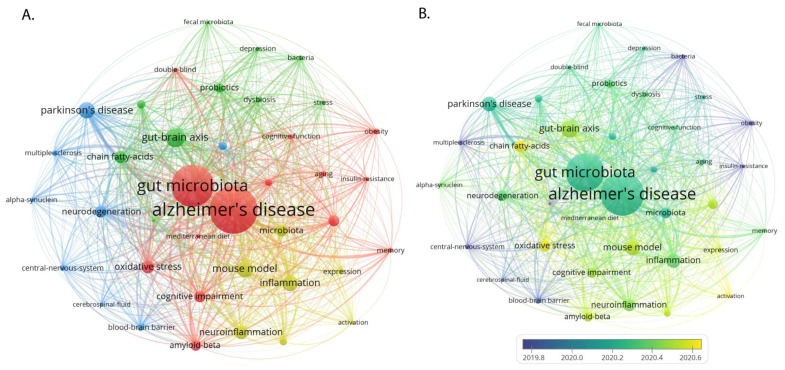
Analysis of research hotspots on the gut-brain axis and psychiatric disorders, including (**A**) a network visualization map of co-occurring keywords and (**B**) an overlay visualization map of keywords by year. (**A**) Keywords in different fields are represented by dots of various colors. The size of the color indicates the frequency of occurrence. (**B**) The color from blue to yellow indicates their chronological order of occurrence. The closer the color is to yellow, the more recent the research is for that particular keyword.

Of the 5165 keywords identified from the 1170 articles on gut microbiota and AD, 71 keywords that appeared more than 30 times were analyzed (Figure 6A). The most frequent keywords were “Alzheimer’s disease” (878), “Gut microbiota” (751), and “gut-brain axis” (296).

These keywords were organized into four clusters that highlight specific areas of interest.

Cluster 1 (red) focuses on nutrition metabolism and neurodegenerative diseases. It includes keywords such as “amyloid-beta”, “cognitive function”, “obesity”, “Alzheimer’s disease”, “insulin-resistance”, and “Mediterranean diet”, indicating a line of research exploring dietary and metabolic influences on AD and other neurodegenerative conditions. Cluster 2 (green) is centered on fecal composition and the role of gut microbiota. Keywords such as “fecal microbiota”, “chain fatty-acids”, and “probiotics” suggest a focus on understanding how changes in gut microbiota, possibly influenced by diet or probiotics, can impact AD progression. Cluster 3 (deep blue) involves CNS topics such as “alpha-synuclein”, “blood-brain barrier”, and “cerebrospinal fluid”. This suggests a focus on the direct connections and communication between the brain and gut, and how these may influence or be influenced by AD. Cluster 4 (yellow) concentrates on pathophysiological reactions, including “expression”, “activation”, “microglia”, and “neuroinflammation”, highlighting research into the molecular and cellular mechanisms involved in AD, especially those involving inflammation and immune cells.

The analysis of the past 5 years shows the most studied areas were “chain fatty-acids”, “oxidative stress”, and “gut-brain axis” (Figure 6B). This could suggest an increasing interest in understanding the role of diet and metabolic processes (chain fatty-acids), the damage caused by reactive oxygen species (oxidative stress), and the gut-brain communication pathway (gut-brain axis) in the context of AD.

#### 3.6.2. Keywords with the Strongest Citation Bursts

**Figure 7:**
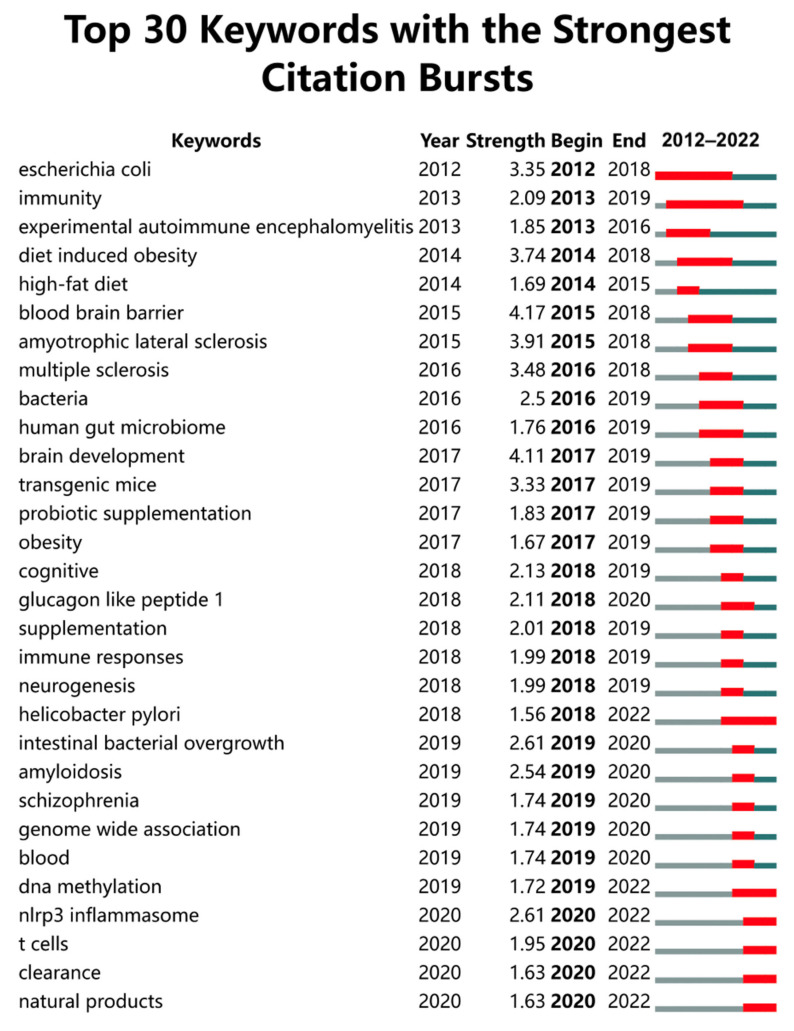
The top 30 keywords with the strongest citation bursts from 2012 to 2022. The progression of keyword emergence from its inception to its formation depicted by a prominent red horizontal line. Years characterized by relatively lower usage of the keyword are represented by gray horizontal line.

Figure 7 provides an interesting perspective on the temporal evolution of the research focus within the field of gut microbiota and AD. It highlights the top 30 keywords with the strongest citation bursts, indicating periods of intense research activity around and interest in these topics. “*Escherichia coli*” and “Immunity” had sustained interest during 2012–2018 and 2013–2019, respectively. This suggests that during this period, the role of specific gut microbiota (such as *E. coli*) and the broader immune response in AD were key research focal points. This could be related to investigations into how alterations in gut microbiota composition, such as an overgrowth of *E. coli*, could influence immune responses and contribute to neurodegenerative processes seen in AD.

The shift in focus in recent years towards keywords such as “Helicobacter pylori” (2018–2022), “DNA methylation” (2019–2022), “NLRP3 inflammasome” (2020–2022), “T cells” (2020–2022), “Clearance” (2020–2022), and “Natural products” (2020–2022) indicate evolving research priorities. “Helicobacter pylori” probably indicates an intriguing exploration of the potential role of this bacteria, commonly associated with gastric ulcers, in the development of AD. The term “DNA methylation” alludes to an epigenetic mechanism that can impact gene expression and may be influenced by gut microbiota. This suggests a research focus on unraveling the molecular mechanisms that connect gut microbiota with AD. Burst words “NLRP3 inflammasome” and “T cells” shed light on a sustained interest in comprehending the involvement of the immune system, particularly inflammation and specific immune cell types, in AD. The term “clearance” likely pertains to the elimination of toxic substances or waste products from the brain, a process that has been linked to AD when impaired. Finally, “natural products” suggests a specific emphasis on exploring potential natural or dietary interventions to modulate gut microbiota or other processes. This signifies a keen interest in investigating interventions that leverage the power of natural compounds for potential therapeutic strategies.

These emerging areas represent potential future research directions in this field. They suggest an increasingly sophisticated understanding of the complex relationships between the gut microbiota, immune system, epigenetic changes, and clearance mechanisms, and how these could be manipulated, to prevent, manage, or treat AD.

## 4. Discussion

Over the past decade, there has been an upsurge in research investigating the connection between gut microbiota and AD, underlining the evolving paradigm in biomedical research. Our study provides a comprehensive overview of this surge and identifies the most significant contributors in terms of countries, institutions, and journals. Between 2012 and 2022, a total of 1170 publications were identified, revealing an increasing trend in this emergent area of research, propelled by significant initiatives such as the second phase of the Integrated Human Microbiome Project [28], and rising global concern for dementia as highlighted in the Alzheimer’s Disease International’s Global Dementia Report of 2022 [29].

From our data, several countries stand out as significant contributors to this research field. China, the USA, and Italy appear as the most productive, with China’s high output likely reflecting its government’s commitment to Alzheimer’s research and microbial studies, supported by the National Natural Science Foundation of China and the Healthy China Action plan (2019–2030) [30]. Additionally, the application of traditional Chinese medicine in studying AD’s underlying mechanisms and prevention cannot be underestimated [31,32].

Our study found that the Journal of Alzheimer’s Disease, Nutrients, and the International Journal of Molecular Science were the most productive journals in this field. However, these and the other top seven journals combined only accounted for less than 30% of the total publications identified. Therefore, over 70% of the research in this area has been published across a wide range of other journals. This distribution could be an indication of the interdisciplinary nature of this research field, transcending beyond a single area of focus. In this digital age, the accessibility and searchability of research content have been enhanced, and researchers are not necessarily restricted to specific journals for sourcing their information. While analyzing the productivity of different journals can provide a broad perspective on the most reputable journals in a particular field, it may not necessarily guide researchers in their choice of resources.

Shanghai Jiaotong University has been an early and significant contributor to the field of gut microbiota and AD, accounting for approximately 5.1% of the publications in our dataset. It is worth noting that six out of the top ten contributors were Chinese institutions. The collaboration network analysis revealed that Shanghai Jiaotong University has been actively collaborating with a variety of domestic and foreign institutions. Similarly, both the Chinese Academy of Sciences and Harvard Medical School have established broad collaborations in this field. The author co-occurrence analysis suggests a pattern of strong internal cohesion within different author groups, with more limited intergroup collaborations. Notably, these findings only indicate the number of contributions and do not necessarily reflect the quality or impact of the research conducted.

The most cited articles in a field are often considered the most valuable and impactful findings that can influence further research. Our analysis found that the article “The Microbiota-Gut-Brain Axis” (2019), published in Physiological Reviews, was the most cited article with a total of 1212 citations by the end of the inception day. This article outlines the applications of the microbiota-gut-brain axis in the study of psychiatric, neurodevelopmental, and neurodegenerative diseases, lists the various pathways by which the microbiota and the brain communicate, and explains the link between the gut microbiota and many diseases including AD [19]. The second most cited article, “What is the Healthy Gut Microbiota Composition? A Changing Ecosystem across Age, Environment, Diet, and Diseases” (2019), highlights the close interrelationship between gut microbiota changes and disease [20]. This paper underscores the importance of maintaining a healthy balance of gut microbiota in individuals.

Our hotspot analysis reveals frequent mentions of oxidative stress and neuroinflammation in the corpus of literature examining the connection between gut microbiota and AD. While we must stress that the frequency of keywords does not establish causal relationships or imply definitive biological importance, it does suggest these are active areas of exploration in this field. Research has indicated that cellular senescence can affect immune function, which in turn can cause damage to the blood-brain barrier [33,34,35]. A notable example of a research focus in this area is the nucleotide-binding domain, leucine-rich–containing family, pyrin domain–containing-3 (NLRP3) inflammasome, on which recent studies have suggested could have a role in Alzheimer’s pathogenesis [36]. In terms of interventions, gut microbiota regulators and NSAIDs with high safety profiles are being examined as potential therapeutic options [24,37]. However, it is essential to note that these are exploratory areas of research, and their clinical utility remains to be firmly established. Additionally, natural compounds such as ChEIs extracted from herbal medicines have emerged as potential therapeutic agents [38].

There has been a discernible shift in the research focus from separate investigations of microbiota and dementia towards a more integrated view, with particular emphasis on the potential influence of short-chain fatty acids (SCFA) on AD. While our analysis has identified common keywords such as ‘obesity’, ‘lipid’, and ‘inflammation’. Once again, the presence of these terms does not constitute proof of their biomedical significance in the disease process. Future in-depth studies are needed to validate their role in the disease’s pathogenesis. The gut microbiota’s potential role in Alzheimer’s disease has been explored in several studies, with some suggesting that modifications in gut microbiota could potentially prevent AD [39]. However, it is important to clarify that these are early-stage findings, and more rigorous investigations are needed to ascertain their therapeutic value.

It is important to acknowledge that our investigation has limitations, as per the bibliometric analysis guidelines. Ideally, a single database should be used, as recommended by Donthu et al. (2021) [40]. In this study, we only included publications from the WoS, the largest biomedical database commonly used for bibliometric analysis. Since we employed multiple analysis software, such as Vosviewer, a bibliometric website, and Citespace, it may result in data loss and some systematic errors in the analysis process. However, this does not significantly impact the general trends in this research area. In interpreting our findings, this type of analysis is effective in determining the volume and progression of research on a specific topic, it does not differentiate between the quality of individual studies. Consequently, our results primarily reflect the quantity, not the quality, of published studies on the interaction between AD and gut microbes. Therefore, the frequency of specific terms or research trends identified in our study should not be seen as a definitive indicator of their biomedical importance or priority in this field. Rather, they serve to highlight areas of active research and broad interest within the scientific community. In light of these limitations, we would like to mention that the word burst analysis has inherent limitations in fast-paced research fields. The conclusions drawn from our results are based on past and present trends, which may not necessarily continue in the future. Future advancements in the field may alter the trajectory of research and shift the focus to different areas.

In conclusion, research on the relationship between gut microbiota and AD has rapidly progressed in the past decade. Recent studies have highlighted the importance of the gut-brain axis, short-chain fatty acids, oxidative stress, and neuroinflammation in AD. Remarkably, gut microbial targeted therapy has emerged as a promising new approach to treat AD, indicating a potential paradigm shift in the management of AD.

## Figures and Tables

**Table 1:** Number of annual publications on brain-gut axis and AD (N = 1170).

Year	NP	Percent (%)	TC/Y	TC
2012	2	0.2	3.5	76
2013	7	0.6	6.9	486
2014	6	0.5	6.8	368
2015	14	1.2	8.9	1001
2016	25	2.1	15.1	2637
2017	55	4.7	20.5	6772
2018	102	8.7	11.5	5840
2019	134	11.5	14.5	7761
2020	173	14.8	8.5	4422
2021	259	22.1	4.8	2476
2022	393	33.6	1.4	531

Note: NP: Number of Publication; TC/Y: Average per Year Total Citations; TC: Total Citations; TC/Y: Average per Year Total Citations.

**Table 2:** Top 10 most productive countries of corresponding authors on gut microbiota and Alzheimer’s Disease.

SCR	Country	NP	Percent (%)	TC	SCP	MCP	MCP_Ratio
1	China	374	40.7	8268	316	58	0.155
2	USA	208	22.6	9544	146	62	0.298
3	Italy	90	9.8	5159	65	25	0.278
4	Korea	50	5.4	1206	46	4	0.08
5	Spain	40	4.4	1301	29	11	0.275
6	Australia	34	3.7	1070	22	12	0.353
7	Japan	33	3.6	674	26	7	0.212
8	Canada	31	3.4	1632	18	13	0.419
9	Poland	31	3.4	1019	28	3	0.097
10	India	28	3.1	462	20	8	0.286

Notes: SCR: Standard Competition Ranking; NP: Number of Publication; TC: Total Citations; SCP: Single Country Publications; MCP: Multiple Country Publications.

**Table 3:** Top 10 most productive journals on gut microbiota and Alzheimer’s Disease.

Sources	NP	Percent (%)	IF (2021)	Category (JCR)
Journal of Alzheimers Disease	57	4.9	4.16	Neurosciences (Q2)
Nutrients	50	4.3	6.706	Nutrition and Dietetics (Q1)
International Journal of Molecular Science	49	4.2	6.208	Biochemistry and Molecular Biology (Q2); Chemistry, Multidisciplinary (Q2)
Frontiers in Aging Neuroscience	46	3.9	5.702	Neurosciences (Q2); Geriatrics and Gerontology (Q2)
Frontiers in Neuroscience	31	2.7	5.152	Neurosciences (Q2)
Scientific Reports	26	2.2	4.997	Multidisciplinary Sciences (Q1)
Frontiers in Immunology	21	1.8	8.787	Immunology (Q1)
Frontiers in Pharmacology	16	1.4	5.988	Phamacology and Phamacy (Q1)
Cells	13	1.1	7.666	Cell Biology (Q2)
Brain Behavior and Immunity	12	1.0	19.227	Neurosciences (Q1); Immunology (Q1); Psychiatry (Q1)

Note: NP, Number of publications; IF: Impact Factor; JCR: Journal Citation Reports.

**Table 4:** Top 10 most active authors contributing to research on gut microbiota and Alzheimer’s Disease.

SCR	Author (N = 4683)	H-Index	TC	NP	Institution (N = 1940)	NP	Percent (%)
1	Wang Y	13	770	31	Shanghai Jiaotong University	66	5.6
2	Zhang X	13	1504	28	University of Kentucky	60	5.1
3	ZhangY	7	189	26	Zhejiang University	59	5.0
4	Li Y	11	354	25	Louisiana University	49	4.2
5	Wang X	10	790	25	Fudan University	41	3.5
6	Liu Y	8	184	22	Soochow University	41	3.5
7	Chen Y	10	487	17	Capital Medical University	39	3.3
8	Liu J	8	788	17	Peking University	38	3.3
9	Zhang L	7	566	17	University College Cork	37	3.2
10	Zhao Y	7	419	17	Central South University	36	3.1

Note: SCR: Standard Competition Ranking; NP: Number of Publication; TC: Total Citations.

**Table 5:** Top 10 most cited articles on gut microbiota and Alzheimer’s Disease.

SCR	Author and Year	Title	Journal (IF-2022)	TC	TC/Y
1	Cryan JF, 2019 [19]	The Microbiota-Gut-Brain Axis	Physiological Reviews (46.513; Q1)	1212	242.4
2	Rinninella E, 2019 [20]	What is the Healthy Gut Microbiota Composition? A Changing Ecosystem across Age, Environment, Diet, and Diseases	Microorganisms (4.926; Q3)	980	196.0
3	Fung TC, 2017 [21]	Interactions between the microbiota, immune and nervous systems in health and disease	Nature Neuroscience (28.771; Q1)	887	126.7
4	Vogt NM, 2017 [10]	Gut microbiome alterations in Alzheimer’s disease	Scientific Reports (4.997; Q3)	809	115.6
5	Hickman S, 2018 [22]	Microglia in neurodegeneration	Nature Neuroscience (28.771; Q1)	635	105.8
6	Cattaneo A, 2017 [23]	Association of brain amyloidosis with pro-inflammatory gut bacterial taxa and peripheral inflammation markers in cognitively impaired elderly	Neurobiology of Aging (5.133; Q2/3)	578	82.6
7	Wang X, 2019 [24]	Sodium oligomannate therapeutically remodels gut microbiota and suppresses gut bacterial amino acids-shaped neuroinflammation to inhibit Alzheimer’s disease progression	Cell Research (46.351, Q1)	445	89.0
8	Akbari E, 2016 [25]	Effect of Probiotic Supplementation on Cognitive Function and Metabolic Status in Alzheimer’s Disease: A Randomized, Double-Blind and Controlled Trial	Frontiers in Aging Neuroscience (5.702; Q2/3)	423	52.9
9	Harach T, 2017 [26]	Reduction of Abeta amyloid pathology in APPPS1 transgenic mice in the absence of gut microbiota	Scientific Reports (4.997; Q3)	418	59.7
10	Jiang C, 2017 [27]	The Gut Microbiota and Alzheimer’s Disease	Journal of Alzheimers Disease (4.160, Q3)	394	56.3

Note: SCR: Standard Competition Ranking; IF: Impact Factor; TC: Total Citations. TC/Y: Average per Year Total Citations.

## Data Availability

Not applicable.

## Supplementary Materials

The following supporting information can be downloaded at: https://www.mdpi.com/article/10.3390/nu15143203/s1, Figure S1: Top 20 countries with the highest productivity of corresponding authors in research on gut microbiota and Alzheimer’s disease.

## References

[1] Alander M., Satokari R., Korpela R., Saxelin M., Vilpponen-Salmela T., Mattila-Sandholm T., von Wright A. (1999). Persistence of colonization of human colonic mucosa by a probiotic strain, Lactobacillus rhamnosus GG, after oral consumption. Appl. Environ. Microbiol..

[2] Schemann M., Neunlist M. (2004). The human enteric nervous system. Neurogastroenterol. Motil..

[3] Clarke G., Grenham S., Scully P., Fitzgerald P., Moloney R., Shanahan F., Dinan T., Cryan J. (2013). The microbiome-gut-brain axis during early life regulates the hippocampal serotonergic system in a sex-dependent manner. Mol. Psychiatry.

[4] McKernan D., Dennison U., Gaszner G., Cryan J., Dinan T. (2011). Enhanced peripheral toll-like receptor responses in psychosis: Further evidence of a pro-inflammatory phenotype. Transl. Psychiatry.

[5] Serino M., Fernández-Real J.M., Fuentes E.G., Queipo-Ortuno M., Moreno-Navarrete J.M., Sánchez A., Burcelin R., Tinahones F. (2013). The gut microbiota profile is associated with insulin action in humans. Acta Diabetol..

[6] (2022). 2022 Alzheimer’s disease facts and figures. Alzheimers Dement..

[7] Rajan K.B., Weuve J., Barnes L.L., McAninch E.A., Wilson R.S., Evans D.A. (2021). Population estimate of people with clinical Alzheimer’s disease and mild cognitive impairment in the United States (2020–2060). Alzheimers Dement..

[8] Erny D., Hrabě de Angelis A.L., Jaitin D., Wieghofer P., Staszewski O., David E., Keren-Shaul H., Mahlakoiv T., Jakobshagen K., Buch T. (2015). Host microbiota constantly control maturation and function of microglia in the CNS. Nat. Neurosci..

[9] Yarandi S.S., Peterson D.A., Treisman G.J., Moran T.H., Pasricha P.J. (2016). Modulatory effects of gut microbiota on the central nervous system: How gut could play a role in neuropsychiatric health and diseases. J. Neurogastroenterol. Motil..

[10] Vogt N.M., Kerby R.L., Dill-McFarland K.A., Harding S.J., Merluzzi A.P., Johnson S.C., Carlsson C.M., Asthana S., Zetterberg H., Blennow K. (2017). Gut microbiome alterations in Alzheimer’s disease. Sci. Rep..

[11] Sochocka M., Donskow-Łysoniewska K., Diniz B.S., Kurpas D., Brzozowska E., Leszek J. (2019). The gut microbiome alterations and inflammation-driven pathogenesis of Alzheimer’s disease—A critical review. Mol. Neurobiol..

[12] Scheltens P., De Strooper B., Kivipelto M., Holstege H., Chetelat G., Teunissen C.E., Cummings J., van der Flier W.M. (2021). Alzheimer’s disease. Lancet.

[13] Kowalski K., Mulak A. (2019). Brain-gut-microbiota axis in Alzheimer’s disease. J. Neurogastroenterol. Motil..

[14] Saji N., Niida S., Murotani K., Hisada T., Tsuduki T., Sugimoto T., Kimura A., Toba K., Sakurai T. (2019). Analysis of the relationship between the gut microbiome and dementia: A cross-sectional study conducted in Japan. Sci. Rep..

[15] Osinska V., Klimas R. (2021). Mapping science: Tools for bibliometric and altmetric studies. Inf. Res. Int. Electron. J..

[16] Darvish H.R. (2018). Bibliometric Analysis using Bibliometrix an R Package. J. Sci. Res..

[17] Grech V., Rizk D.E.E. (2018). Increasing importance of research metrics: Journal Impact Factor and h-index. Int. Urogynecol. J..

[18] Van Eck N.J., Waltman L. (2010). Software survey: VOSviewer, a computer program for bibliometric mapping. Scientometrics.

[19] Cryan J.F., O’Riordan K.J., Cowan C.S.M., Sandhu K.V., Bastiaanssen T.F.S., Boehme M., Codagnone M.G., Cussotto S., Fulling C., Golubeva A.V. (2019). The Microbiota-Gut-Brain Axis. Physiol. Rev..

[20] Rinninella E., Raoul P., Cintoni M., Franceschi F., Miggiano G.A.D., Gasbarrini A., Mele M.C. (2019). What is the Healthy Gut Microbiota Composition? A Changing Ecosystem across Age, Environment, Diet, and Diseases. Microorganisms.

[21] Fung T.C., Olson C., Hsiao E. (2017). Interactions between the microbiota, immune and nervous systems in health and disease. Nat. Neurosci..

[22] Hickman S., Izzy S., Sen P., Morsett L., El Khoury J. (2018). Microglia in neurodegeneration. Nat. Neurosci..

[23] Cattaneo A., Cattane N., Galluzzi S., Provasi S., Lopizzo N., Festari C., Ferrari C., Guerra U.P., Paghera B., Muscio C. (2017). Association of brain amyloidosis with pro-inflammatory gut bacterial taxa and peripheral inflammation markers in cognitively impaired elderly. Neurobiol. Aging.

[24] Wang X., Sun G., Feng T., Zhang J., Huang X., Wang T., Xie Z., Chu X., Yang J., Wang H. (2019). Sodium oligomannate therapeutically remodels gut microbiota and suppresses gut bacterial amino acids-shaped neuroinflammation to inhibit Alzheimer’s disease progression. Cell Res..

[25] Akbari E., Asemi Z., Daneshvar Kakhaki R., Bahmani F., Kouchaki E., Tamtaji O.R., Ali Hamidi G., Salami M. (2016). Effect of Probiotic Supplementation on Cognitive Function and Metabolic Status in Alzheimer’s Disease: A Randomized, Double-Blind and Controlled Trial. Front. Aging Neurosci..

[26] Harach T., Marungruang N., Duthilleul N., Cheatham V., Mc Coy K.D., Frisoni G., Neher J.J., Fåk F., Jucker M., Lasser T. (2017). Reduction of Abeta amyloid pathology in APPPS1 transgenic mice in the absence of gut microbiota. Sci. Rep..

[27] Jiang C., Li G., Huang P., Liu Z., Zhao B. (2017). The Gut Microbiota and Alzheimer’s Disease. J. Alzheimers Dis..

[28] The Integrative HMP (iHMP) Research Network Consortium (2019). The Integrative Human Microbiome Project. Nature.

[29] Gauthier S., Webster C., Servaes S., Morais J., Rosa-Neto P. World Alzheimer Report 2022. https://www.alzint.org/resource/world-alzheimer-report-2022/.

[30] Chen P., Li F., Harmer P. (2019). Healthy China 2030: Moving from blueprint to action with a new focus on public health. Lancet Public Health.

[31] Fang Z., Tang Y., Ying J., Tang C., Wang Q. (2020). Traditional Chinese medicine for anti-Alzheimer’s disease: Berberine and evodiamine from Evodia rutaecarpa. Chin. Med..

[32] Pei H., Ma L., Cao Y., Wang F., Li Z., Liu N., Liu M., Wei Y., Li H. (2020). Traditional Chinese Medicine for Alzheimer’s Disease and Other Cognitive Impairment: A Review. Am. J. Chin. Med..

[33] Coder B., Wang W., Wang L., Wu Z., Zhuge Q., Su D.M. (2017). Friend or foe: The dichotomous impact of T cells on neuro-de/re-generation during aging. Oncotarget.

[34] Kebir H., Kreymborg K., Ifergan I., Dodelet-Devillers A., Cayrol R., Bernard M., Giuliani F., Arbour N., Becher B., Prat A. (2007). Human TH17 lymphocytes promote blood-brain barrier disruption and central nervous system inflammation. Nat. Med..

[35] Mou Y., Du Y., Zhou L., Yue J., Hu X., Liu Y., Chen S., Lin X., Zhang G., Xiao H. (2022). Gut Microbiota Interact with the Brain Through Systemic Chronic Inflammation: Implications on Neuroinflammation, Neurodegeneration, and Aging. Front. Immunol..

[36] Zhang Y., Wang L., Lv Y., Jiang C., Wu G., Dull R.O., Minshall R.D., Malik A.B., Hu G. (2019). The GTPase Rab1 Is Required for NLRP3 Inflammasome Activation and Inflammatory Lung Injury. J. Immunol..

[37] Wang J., Tan L., Wang H.F., Tan C.C., Meng X.F., Wang C., Tang S.W., Yu J.T. (2015). Anti-inflammatory drugs and risk of Alzheimer’s disease: An updated systematic review and meta-analysis. J. Alzheimers Dis..

[38] Jia J.Y., Zhao Q.H., Liu Y., Gui Y.Z., Liu G.Y., Zhu D.Y., Yu C., Hong Z. (2013). Phase I study on the pharmacokinetics and tolerance of ZT-1, a prodrug of huperzine A, for the treatment of Alzheimer’s disease. Acta Pharmacol. Sin..

[39] Marizzoni M., Cattaneo A., Mirabelli P., Festari C., Lopizzo N., Nicolosi V., Mombelli E., Mazzelli M., Luongo D., Naviglio D. (2020). Short-Chain Fatty Acids and Lipopolysaccharide as Mediators Between Gut Dysbiosis and Amyloid Pathology in Alzheimer’s Disease. J. Alzheimers Dis..

[40] Donthu N.K.S., Mukherjee D., Pandey N., Lim W.M. (2021). How to conduct a bibliometric analysis: An overview and guidelines. J. Bus. Res..

