# Supplementary material for: Mapping Research Trends and Hotspots in the Link between Alzheimer’s Disease and Gut Microbes over the Past Decade: A Bibliometric Analysis
Source: Nutrients. 2023 Jul 19;15(14):3203. doi: 10.3390/nu15143203 (PMC10383611; doi:10.3390/nu15143203)

Figure S1. Top 20 countries with the highest productivity of corresponding authors in research on gut microbiota and Alzheimer's disease.

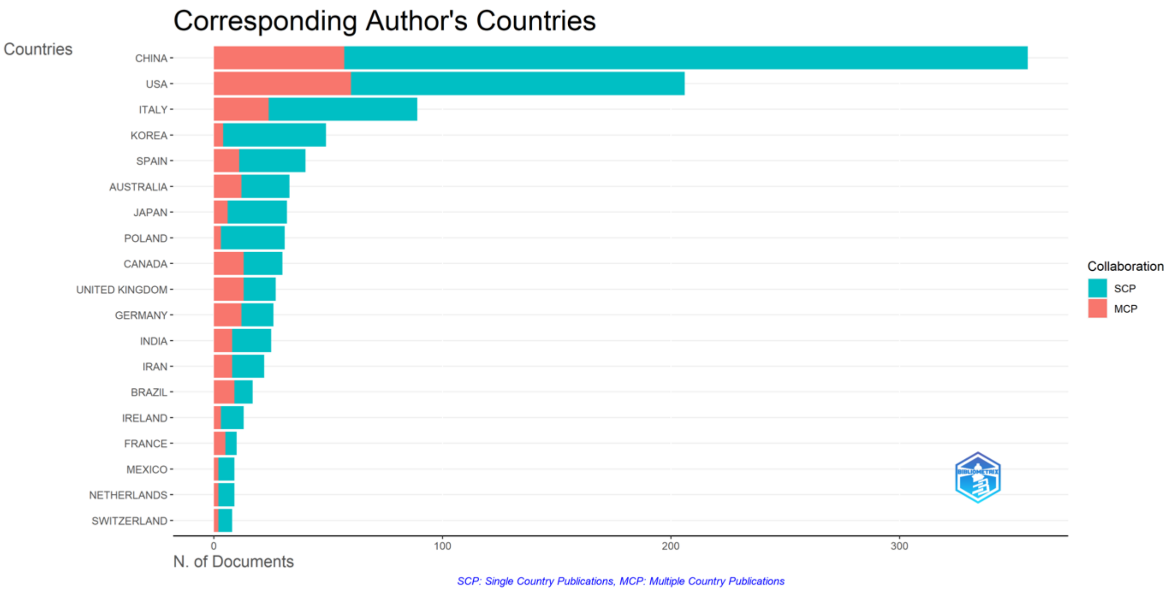

Supplement: Supplementary file 1 [file nutrients-15-03203-s001.zip › nutrients-2457938-supplementary.pdf]
